# Molecular Characterization of *Haemaphysalis* Species and a Molecular Genetic Key for the Identification of *Haemaphysalis* of North America

**DOI:** 10.3389/fvets.2020.00141

**Published:** 2020-03-13

**Authors:** Alec T. Thompson, Kristen Dominguez, Christopher A. Cleveland, Shaun J. Dergousoff, Kandai Doi, Richard C. Falco, Telleasha Greay, Peter Irwin, L. Robbin Lindsay, Jingze Liu, Thomas N. Mather, Charlotte L. Oskam, Roger I. Rodriguez-Vivas, Mark G. Ruder, David Shaw, Stacey L. Vigil, Seth White, Michael J. Yabsley

**Affiliations:** ^1^Southeastern Cooperative Wildlife Disease Study, Department of Population Health, College of Veterinary Medicine, University of Georgia, Athens, GA, United States; ^2^Center for the Ecology of Infectious Diseases, Odum School of Ecology, University of Georgia, Athens, GA, United States; ^3^Agriculture and Agri-Food Canada, Lethbridge Research and Development Centre, Lethbridge, AB, Canada; ^4^Laboratory of Wildlife Medicine, Nippon Veterinary and Life Science University, Musashino, Japan; ^5^New York State Department of Health, Louis Calder Center, Fordham University, Armonk, NY, United States; ^6^Vector and Waterborne Pathogens Research Group, College of Science, Health, Engineering and Education, Murdoch University, Murdoch, WA, Australia; ^7^Public Health Agency of Canada, National Microbiology Laboratory, Winnipeg, MB, Canada; ^8^Key Laboratory of Animal Physiology, Biochemistry and Molecular Biology of Hebei Province, College of Life Sciences, Hebei Normal University, Shijiazhuang, China; ^9^Center for Vector-Borne Diseases, University of Rhode Island, Kingston, RI, United States; ^10^Campus of Biology and Agricultural Sciences, Department of Veterinary Medicine and Animal Husbandry, National Autonomous University of Yucatan, Merida, Mexico; ^11^Warnell School of Forestry and Natural Resources, University of Georgia, Athens, GA, United States

**Keywords:** *Haemaphysalis*, Asian longhorned tick, PCR-RFLP, molecular key, invasive, phylogenetic

## Abstract

*Haemaphysalis longicornis* (Acari: Ixodidae), the Asian longhorned tick, is native to East Asia, but has become established in Australia and New Zealand, and more recently in the United States. In North America, there are other native *Haemaphysalis* species that share similar morphological characteristics and can be difficult to identify if the specimen is damaged. The goal of this study was to develop a cost-effective and rapid molecular diagnostic assay to differentiate between exotic and native *Haemaphysalis* species to aid in ongoing surveillance of *H. longicornis* within the United States and help prevent misidentification. We demonstrated that restriction fragment length polymorphisms (RFLPs) targeting the 16S ribosomal RNA and the cytochrome *c* oxidase subunit I (*COI*) can be used to differentiate *H. longicornis* from the other *Haemaphysalis* species found in North America. Furthermore, we show that this RFLP assay can be applied to *Haemaphysalis* species endemic to other regions of the world for the rapid identification of damaged specimens. The work presented in this study can serve as the foundation for region specific PCR-RFLP keys for *Haemaphysalis* and other tick species and can be further applied to other morphometrically challenging taxa.

## Introduction

Ticks are important vectors of numerous pathogens for humans and animals throughout the world. The accurate identification of tick species, often through morphological characteristics, is of great importance to public and veterinary health and for the control of associated tick-borne diseases. One of the key morphologic characteristics used to differentiate ixodid ticks are the mouthparts (1, 2), but often these features are damaged during tick collection, making species identification difficult or impossible.

One tick of recent importance in the United States (USA) is *Haemaphysalis longicornis*, the Asian longhorned tick. Native to East Asia, *H. longicornis* has become invasive in multiple regions of the world, largely due to its parthenogenetic reproduction, broad habitat use, and high diversity of avian and mammalian hosts (3–5). In the native range of *H. longicornis*, numerous bacterial, protozoal, and viral pathogens have been detected within this tick, including *Anaplasma* spp., *Borrelia burgdorferi, Theileria* spp., *Babesia* spp., and spotted fever group *Rickettsia* (6–12). Many of these pathogens are zoonotic in nature, thus this tick is of significant importance to both human and animal health. Recently, *H. longicornis* has also been confirmed as the vector for an emerging phlebovirus that causes Severe Fever with Thrombocytopenia Syndrome which can have mortality rates up to 40% (13–15). In North America, a pathogen of significant importance is *B. burgdorferi*, causative agent of Lyme disease. A recent laboratory study showed that *H. longicornis* is not a competent vector for the B31 strain of *B. burgdorferi* (16). In addition, field studies in New York, USA have not found *H. longicornis* on *Peromyscus* spp., the primary reservoir of *B. burgdorferi* (17). Collectively, these studies suggest that the transmission of this bacterial pathogen by *H. longicornis* may be limited. An additional concern with the introduction of an exotic disease vector species is the introduction of exotic pathogens that may be transmitted in its native range. In 2017, the exotic *Theileria orientalis* Ikeda genotype, historically known to be vectored by *H. longicornis*, was determined to be the cause of a mortality event in beef cattle in Virginia, USA (18). In addition, tick burdens on infested hosts can become very high leading to decreased production, growth, and in some cases death as a result of exsanguination causing a concern for the agricultural industry and some wildlife species (19–21). While the potential risks associated with the introduction of exotic *H. longicornis* are great, there is still much work that needs to be done to understand the implications this tick poses to human and animal health within the United States.

*Haemaphysalis longicornis* was first confirmed in the United States in New Jersey on a sheep in late 2017 (22). However, subsequent investigations of archived specimens revealed that *H. longicornis* collected as early as 2010 had been previously misidentified as the native rabbit tick, *Haemaphysalis leporispalustris* (5). With the introduction of *H. longicornis*, there are now four *Haemaphysalis* species known in North America: *H. leporispalustris*, found throughout the Americas and primarily infesting lagomorphs (23, 24); *Haemaphysalis juxtakochi* ranging throughout the Neotropics with cervids or other larger mammals as primary hosts, though it has been found parasitizing migratory neotropical birds (25–27); and *Haemaphysalis chordeilis*, sporadically collected from avian species throughout the United States and Canada (28, 29). These ticks all have wide and, in some places, overlapping distributions.

To aid in the understanding and management of the exotic *H. longicornis*, extensive work has been conducted on the natural history and spread of this tick within the United States (16, 17, 22, 30–32). These studies largely rely on the quick and accurate identification of *H. longicornis* using key morphological features found on the mouth parts (1). However, identification of *Haemaphysalis* ticks, both native and exotic, is difficult if the specimen's mouthparts are damaged during removal from a host. In these cases, molecular confirmation is needed to identify the ticks to the species level. This process can be expensive and time-consuming. Previous studies have shown that a restriction fragment length polymorphism (RFLP) assay is a more rapid and cost-effective method of distinguishing between arthropod vector species (33–36). Our aim was to develop a RFLP molecular assay that could accurately distinguish *H. longicornis* from *Haemaphysalis* species present in North America, as well as from other known *Haemaphysalis* spp. distributed globally.

## Materials and Methods

### Sample Collection

*Haemaphysalis longicornis* and *H. leporispalustris* from the United States were collected through a variety of methods as described by Beard et al. (5). Specimens or DNA of *H. longicornis* (from Australia and China), *H. juxtakochi* (from Mexico), and *H. chordeilis* (from Canada) were collected by collaborators as described in previous studies and through a citizen science program (tickspotters.org) (26, 37–39). Some *Haemaphysalis* spp. endemic to Japan were collected as described by Doi et al. (39), and *Haemaphysalis leachi* was collected as part of an ongoing canine health survey from the Sarh region of Chad, Africa. All ticks were stored in 70–100% ethanol and morphological identification was done with dissecting and compound light microscopy using dichotomous keys to distinguish between the species when possible (1–3, 28, 40, 41).

### Sample Preparation

Ticks collected during this study were bisected and genomic DNA was extracted using a commercial extraction kit (DNeasy® Blood and Tissue Kit, Qiagen, Hilden Germany) following the manufacturer's protocol. The 16S rRNA and cytochrome *c* oxidase subunit 1 (*COI*) genes were targeted for PCR amplification (Table 1). PCR products were visualized on 2% agarose gels stained with GelRed (Biotium, Hayward, California). Amplicons were purified using the QIAquick gel extraction kit (Qiagen) and submitted for bi-directional sequencing at the Genewiz Corporation (South Plainfield, NJ). Chromatograms were analyzed using Geneious R11 (Auckland, New Zealand, https://www.geneious.com). Sequences of unique PCR-RFLPs obtained in this study were deposited in GenBank (accession numbers MN661147-MN661151, MN663150-MN663156, MN991269, and MN994495).

**Table 1:** PCR protocols, gene targets, and restriction enzymes used to obtain 16S rRNA and cytochrome *c* oxidase subunit 1 (*COI*) gene sequences and RFLPs for *Haemaphysalis* spp.

**Gene target**	**Primers**	**Length (bp)**	**References**
16S rRNA	16S-Forward (5′-TTAAATTGCTGTRGTATT-3′) 16S-Reverse (5′-CCGGTCTGAACTCASAWC-3′)	438	(42)
	Restriction Enzyme: *DraI* (5′-TTT∧AAA-3′)		
*COI*	Cox1-F (5′-GGAACAATATATTTAATTTTTGG-3′) CoxI-R (5′-ATCTATCCCTACTGTAAATATATG-3′)	849	(43)
	COI-F (5′-ATCATAAAKAYHTTGG-3′) COI-R (5′-GGGTGACCRAARAAHCA-3′)	691	(42)
	LCO1490 (5′-GGTCAACAAATCATAAAGATATTGG-3′) HCO2198 (5′-TAAACTTCAGGGTGACCAAAAAATCA-3′)	710	(44)
	Restriction Enzyme: *AluI* (5′-AG∧CT-3′)		

For *COI*, three primer pairs were used (Table 1). Primers COI-F/COI-R (691 bp amplicon) and LCO1490/HCO2198 (709 bp amplicon) amplify the same region, with LCO1490/HCO2198 having a 10bp overhang on either side of the COI-F/COI-R primer binding region. As a result, subsequent PCR-RFLP patterns are nearly identical with either set. Additionally, a larger segment of the *COI* gene (820 bp amplicon) was examined using the primer pair Cox1-F/cox1-R. These primers amplify an additional 163bp segment of the *COI* gene that was not already obtained by the previous two primer pairs. This was done to determine if more distinguishable PCR-RFLP patterns may exist between certain *Haemaphysalis* spp. Unfortunately, not all *Haemaphysalis* spp. collected during this study amplified with any of the three *COI* primers tested; however, this inconsistent amplification of the *COI* gene has been documented previously with other genera of ticks (42).

### Restriction Length Polymorphism Assay

All DNA sequences of *H. longicornis, H. leporispalustris, H. juxtakochi*, and *H. chordeilis* collected during this study were aligned and screened with commercially available restriction enzymes to determine candidates to use for the PCR-RFLP assay. *DraI* and *AluI* restriction enzymes (ThermoFisher Scientific, Waltham, MA) were deemed appropriate and used for digestion of the 16S and *COI* gene regions, respectively (Table 1). The manufacturer's protocols were followed for digestion of PCR products. Digested DNA fragments were visualized with gel electrophoresis using 4% agarose gels stained with GelRed to allow for better separation of fragments and to effectively differentiate between the species of ticks from the *Haemaphysalis* genus.

### Bioinformatic Analysis of 16S and COI *Haemaphysalis* spp. Cut Patterns and Phylogenetic Analysis

For the 16S analysis, the query “*Haemaphysalis* 16S” in GenBank returned a total of 1,217 sequences. After filtering for overlapping tick gene sequences and excluding pathogens and endosymbionts isolated from *Haemaphysalis* spp., excess regions were trimmed and 184 *Haemaphysalis* tick 16S gene sequences that overlapped with our amplified region (~438 bp) remained for *in silico* RFLP cut pattern analysis (Table 2). Remaining sequences were aligned, and after artificial digestion with *DraI* through Geneious R11, 16S PCR-RFLP cut patterns were compared with other *Haemaphysalis* spp. Similarly, for the *COI* analysis, two search queries, “*Haemaphysalis* cytochrome *c* oxidase subunit I” and “*Haemaphysalis* COI”, were used to obtain 594 sequences for this gene target. After removing endosymbiont and pathogen sequences, duplicate identical sequences, and trimming excess regions, 124 sequences were available for analysis (Table 3). Because multiple primer sets were used with one amplifying a longer region, sequences included in the study were split into two groups based on amplicon length (~680 and ~820 bp). Amplicons of the two lengths were digested with *AluI* in Geneious R11 for comparison of the PCR-RFLP cut pattern comparisons.

**Table 2:** The 16S rRNA (438bp) sequences from *Haemaphysalis* spp. analyzed for PCR-RFLP patterns.

**Species**	**Endemic regions**	**Sequences analyzed/New sequences from current study**	**No. of PCR-RFLP patterns**	**Representative sequences**
*H. aborensis*	S. Asia	1/0	1	KC170735
*H. asiatica*	E. Asia	1/0	1	KC170734
*H. bispinosa*	Asia/Oceania	18/0	1	KT428017
*H. campanulata*	E. Asia	3/0	1	AB819170
*H. chordeilis*	N. America	1/1	1	MN994495
*H. concinna*	Asia/Europe	7/0	1	AB819171
*H. cornigera*	S. Asia	2/1	1	AB819174
*H. doenitzi*	Asia/Australia	1/0	1	JF979402
*H. elliptica*	S. Africa	4/0	1	HM068956
*H. erinacei*	Asia/Europe	3/0	2	KR870975/KU183521
*H. flava*	E. Asia	5/1	1	KX450279
*H. formosensis*	E. Asia	3/1	1	AB819194
*H. hystricis*	Asia	23/0	1	KC170733
*H. inermis*	Asia/Europe	1/0	1	U95872
*H. japonica douglasi*	E. Asia	2/0	1	AB819176
*H. japonica*	E. Asia	2/0	1	AB819200
*H. juxtakochi*	N. America/S. America	16/11	2	MH513303/MN661147
*H. kitaokai*	E. Asia	15/0	2	MH208539/AB819202
*H. langrangei*	E. Asia	2/0	1	KC170731
*H. leachi*	C. Africa	1/1	1	MN661151
*H. leporispalustris*	N. America	26/26	2	MN661148/MN661149
*H. longicornis*	Asia/Oceania/N. America	85/69	1	MN661150
*H. mageshimaensis*	E. Asia	2/0	1	AB819213
*H. megaspinosa*	E. Asia	6/2	1	AB819214
*H. obesa*	E. Asia	1/0	1	KC170732
*H. parva*	Asia/Europe	1/0	1	KR870977
*H. pentalagi*	E. Asia	2/0	1	AB819218
*H. punctate*	Asia/Europe	5/0	1	KR870978
*H. qinghaiensis*	E. Asia	55/0	1	KJ609201
*H. shimoga*	S. Asia	6/0	1	KC170730
*H*. sp.	China	1/0	1	KU664520
*H. spinigera*	S. Asia	2/0	2	MH044719/MH044720
*H. spinulosa*	Africa	1/0	1	KJ613637
*H. sulcata*	Europe/Asia	2/0	1	KR870979
*H. wellingtoni*	S. Asia	1/0	1	AB819221
*H. yeni*	E. Asia	2/0	1	AB819223
Total		309/113	41	

*H., Haemaphysalis; S. Asia, South Asia; E. Asia, Eastern Asia; S. Africa, South Africa; C. Africa, Central Africa; N. America, North America; S. America, South America*.

**Table 3:** The cytochrome *c* oxidase subunit I (*COI)* (~680 bp) gene sequences from *Haemaphysalis* spp. analyzed for PCR-RFLP patterns.

**Species**	**Endemic regions**	**Sequences analyzed/New sequences from current study**	**No. of PCR-RFLP patterns**	**Representative sequence**
*H. bancrofti*	Oceania (Australia)	1/0	1	NC041076
*H. chordeilis*	N. America	1/1	1	MN991269
*H. concinna*	Asia/Europe	4/0	2	KU170511/NC034785
*H. erinacei*	Asia/Europe	2/0	1	KU364301
*H. flava*	E. Asia	6/0	2	AB075954/HM193865
*H. formosensis*	E. Asia	1/0	1	NC020334
*H. humerosa*	Asia	2/0	1	AF132819
*H. hystricis*	Asia	1/0	1	NC039765
*H. japonica*	E. Asia	1/0	1	NC037246
*H. juxtakochi*	N. America/S. America	6/1	3	KF200077/KF200091/MN663155
*H. leachi*	C. Africa	1/1	1	MN663156
*H. leporispalustris*	N. America	16/13	5	KX360391/MN663151/MN663152/MN663153/MN663154
*H. longicornis*	Asia/Oceania/N. America	104/64	2	AF132820/MG450553
*H. punctata*	Asia/Europe	1/0	1	MH532298
*H. qinghaiensis*	E. Asia	49/0	1	JQ737088
*H. sulcata*	Europe/Asia	4/0	1	MH532303
Total		204/79	28	

*H., Haemaphysalis; E. Asia, Eastern Asia; C. Africa, Central Africa; N. America, North America; S. America, South America*.

Two phylogenetic trees using either the 16S or the *COI* sequences included in this study were generated by aligning sequences using ClustalW and the maximum-likelihood algorithm in MEGA X with the two 16S rRNA and *COI* gene segments of *Rhipicephalus sanguineus* (NC00274 and JX1325) used as the outgroup (45, 46).

## Results

### 16S *Haemaphysalis* spp. Cut Patterns

The query of the 16S gene of *Haemaphysalis* ticks from GenBank and those obtained during this study, yielded a total of 309 sequences from 35 species of proper length (~438 bp) for analysis (Table 2). Of the *Haemaphysalis* ticks with multiple available sequences for comparison, some intraspecies variation was detected for PCR-RFLP cut patterns. *Haemaphysalis* 16S PCR-RFLP patterns were compared with the *Haemaphysalis* spp. endemic to the same regions (**Figure 3**). Only one other *Haemaphysalis* species, *H. asiatica* (KC170734), shared the same PCR-RFLP cut pattern as *H. longicornis* (**Figure 3A**, blue box).

For *Haemaphysalis* spp. endemic to Asia, *H. campanulata* (AB819170), *H. inermis* (U95872), *H. kitaokai* (MH208539), *H. sulcata* (KR870979), and *H. yeni* (AB819223) all share a similar cut pattern (**Figure 3A**, red box). Furthermore, *H. kitaokai* (AB819202), *H. mageshimaensis* (AB819213), and an unidentified *Haemaphysalis* sp. from the Yunnan province of China (KU664520) also share a similar RFLP cut pattern (**Figure 3A**, purple box). *Haemaphysalis* spp. endemic to Africa, Europe, and Oceania all had unique PCR-RFLP cut patterns (**Figures 3B–D**).

### COI *Haemaphysalis* spp. Cut Patterns

The query of the *COI* gene of *Haemaphysalis* ticks from GenBank and those obtained in this study yielded a total of 204 sequences from 15 species (Table 3). More intraspecies *COI* sequence variation was detected compared to the 16S gene target. PCR-RFLP patterns were compared with other *Haemaphysalis* spp. endemic to the same regions (**Figure 4**). Multiple PCR-RFLP cut patterns were detected for *H. longicornis*. Of these, a *H. longicornis* (AF132820) cut pattern was shared with *H. flava* (AB075954; **Figure 4A**, red box), which is endemic throughout Asia.

Additionally, *H. hystricis* (NC039765) and *H. japonica* (NC037246) share the same PCR-RFLP cut pattern (**Figure 4A**, green box). PCR-RFLP cut patterns for *Haemaphysalis* spp. endemic to Europe were all unique (**Figure 2B**). For *Haemaphysalis* spp. endemic to Oceania, specifically Australia, *H. longicornis* (AF132820) shared a cut pattern with an unidentified *Haemaphysalis* sp. collected from a koala (*Phascolarctos cinereus*) from Victoria, Australia (KM821503; **Figure 4C**, red box). Additionally, *H. bancrofti* (NC041076) shared a cut pattern with another unidentified *Haemaphysalis* sp. (KM821502; **Figure 4C**, green box) also collected from a koala from the same region of Australia as previously mentioned. Due to the small number of available *COI* sequences at 820 bp of length (*n* = 17, 11 different *Haemaphysalis* spp.), the larger segment of the gene was excluded from analysis.

### Phylogenetic Analysis

**Figure 1:**
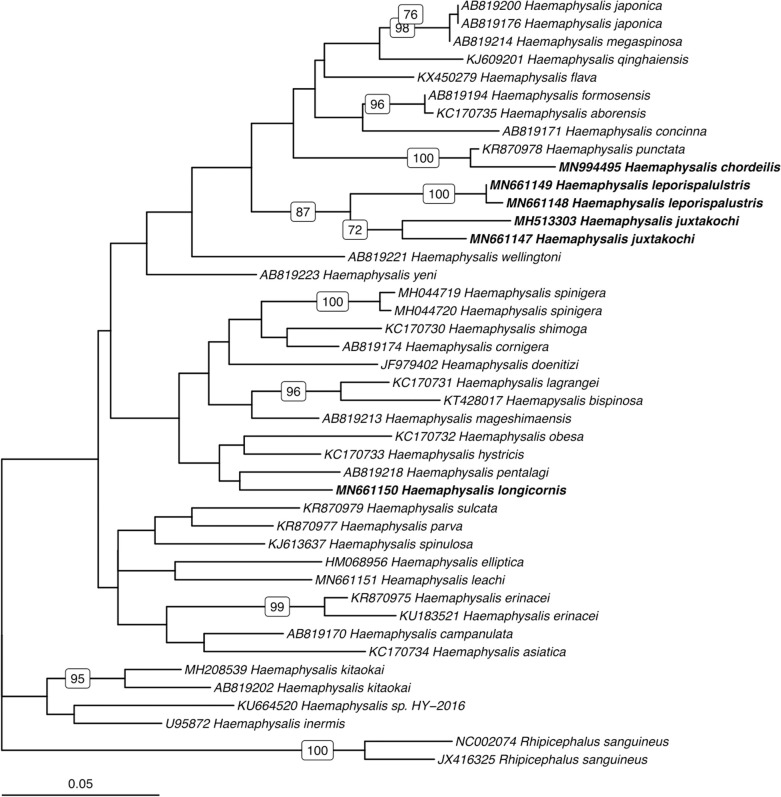
Phylogenetic analysis of the *Haemaphysalis* spp. 16S rRNA gene sequence analyzed for PCR-RFLP. Bolded sequences represent species present in North America. Numbers on branches indicate bootstrap values after 500 iterations, values below 70% were omitted from the tree.

**Figure 2:**
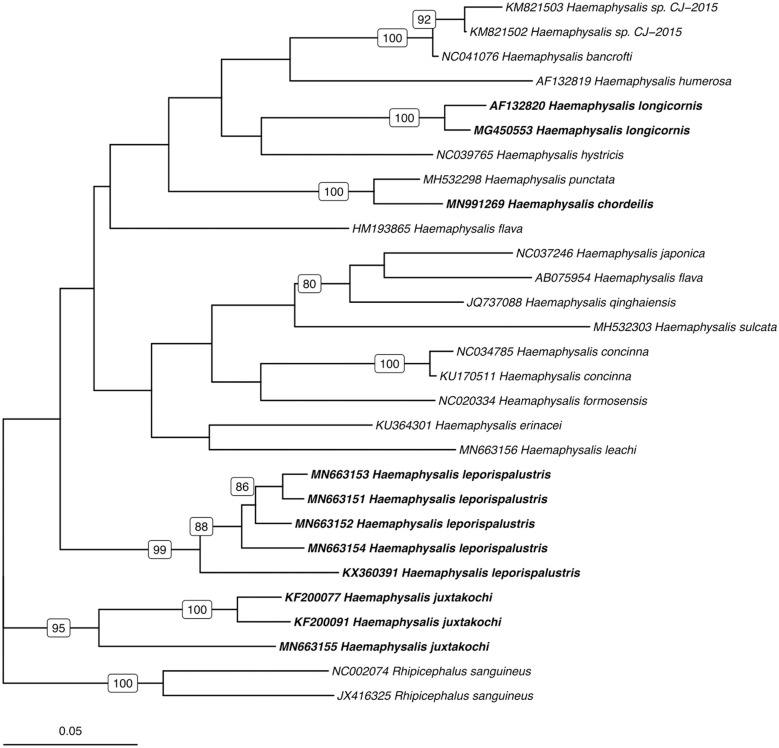
Phylogenetic analysis of the *Haemaphysalis* spp. *COI* gene sequence analyzed for PCR-RFLP. Bolded sequences represent species present in North America. Numbers on branches indicate bootstrap values after 500 iterations, values below 70% were omitted from the tree.

Utilizing the 41 sequences of the unique 16S PCR-RFLPs and the 28 unique from the *COI* PCR-RFLPs (Tables 2, 3), two maximum-likelihood trees were generated for the respective gene targets (Figures 1, 2). There appears to be no clear clustering of tick sequences based on geographic location as *Haemaphysalis* spp. endemic to Asia are dispersed throughout in both the trees. Sequences from both gene targets confirm that *H. longicornis* was genetically distinct from the other *Haemaphysalis* spp. endemic to North America (Figures 1, 2). For the 16S rRNA gene analysis, *H. leporispalustis* and *H. juxtakochi* cluster together but are in two distinct clades in the *COI* analysis due to low bootstrap support for the placement of the *H. juxtakochi* clade (Figures 1, 2). Interestingly, *H. chordeilis* does not group with either North America species with either gene target but instead groups with *H. punctata*, a species native to Eurasia (Figures 1, 2).

### Molecular Key for North American *Haemaphysalis* spp. RFLP Cut Patterns

**Figure 3:**
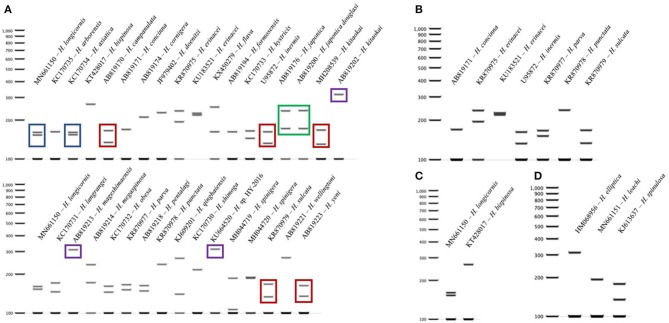
16S PCR-RFLP cut patterns for *Haemaphysalis* spp. endemic to different regions of the world. **(A)** 16S PCR-RFLP cut patterns of *Haemaphysalis* spp. endemic to Asia. Different colored boxes indicate that the PCR-RFLP cut patterns is shared with at least one other *Haemaphysalis* spp.; **(B)** 16S PCR-RFLP cut patterns of *Haemaphysalis* spp. endemic to Europe; **(C)** 16S PCR-RFLP cut patterns of *Haemaphysalis* spp. endemic to Oceania; **(D)** 16S PCR-RFLP cut patterns of *Haemaphysalis* spp. endemic to Africa.

For *Haemaphysalis* spp. endemic to North America, *H. longicornis* had one or more unique PCR-RFLP cut patterns for both gene targets, all of which allow for effective differentiation between the other tick species endemic to this region (**Figure 5**). The inclusion of our sequence data for *Haemaphysalis* spp. present in North America revealed greater diversity than what is currently represented by sequences analyzed from GenBank. For the 16S PCR-RFLP, *H. longicornis* and *H. chordeilis* had a single cut pattern and *H. juxtakochi* and *H. leporispalustris* both had two unique and descriptive PCR-RFLP cut patterns (**Figure 5A**). Unlike the 16S PCR-RFLP, all *Haemaphysalis* ticks of North America had multiple cut patterns for the *COI* PCR-RFLP with the exception of the single *H. chordeilis* sequence included in the study. *H. longicornis* and *H. juxtakochi* both had 2 and 3 unique *COI* PCR-RFLP cut patterns, respectively, and *H. leporispalustris* had 5 cut patterns (**Figure 5B**). A key to species of *Haemaphysalis* ticks of North America was constructed using the 16S PCR-RFLP patterns (Figure 3, See Key to *Haemaphysalis* ticks found in North America); a key for the *COI* PCR-RFLP patterns was not included due to the higher intraspecific variation.

## Discussion

Here we analyze new sequences of several *Haemaphysalis* spp., including the first genetic data for *H. chordeilis*, and describe a molecular assay that can aid in the rapid and accurate identification of any life stage of the four *Haemaphysalis* spp. currently in North America. Ticks collected from hosts are often submitted to diagnostic laboratories damaged or without mouthparts, which are required for specific identification of *Haemaphysalis* spp., so this technique is especially useful. Given the range expansion, or recognized range, of *H. longicornis* in the United States, the need for a rapid response is key and this technique provides an alternative method to accurately identify ticks when morphology becomes unreliable. Furthermore, inclusion of *Haemaphysalis* spp. from other regions of the world suggests that this method can potentially be useful for distinguishing a wide range of species.

**Figure 4:**
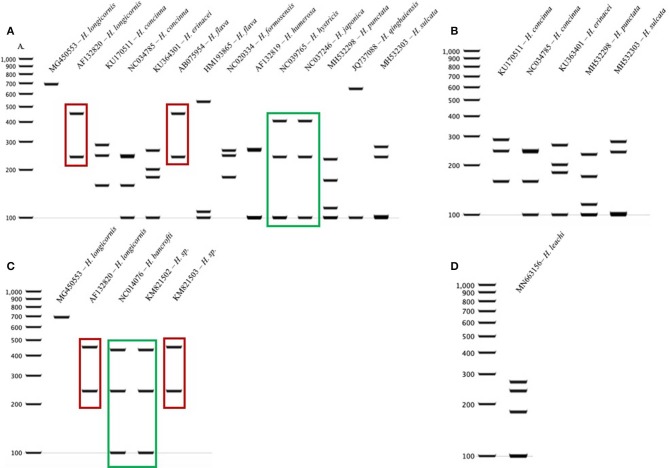
*COI* PCR-RFLP cut patterns for *Haemaphysalis* spp. endemic to different regions of the world. **(A)** *COI* rRNA PCR-RFLP cut patterns of *Haemaphysalis* spp. endemic to Asia. Different colored boxes indicate that the PCR-RFLP cut patterns is shared with at least one other *Haemaphysalis* spp.; **(B)** *COI* PCR-RFLP cut patterns of *Haemaphysalis* spp. endemic to Europe; **(C)** *COI* PCR-RFLP cut patterns of *Haemaphysalis* spp. endemic to Oceania; **(D)** *COI* PCR-RFLP cut patterns of *Haemaphysalis* spp. endemic to Africa.

**Figure 5:**
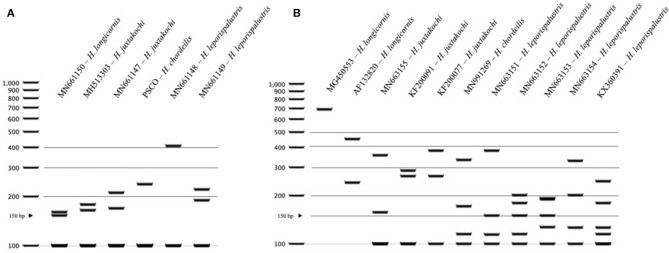
PCR-RFLP cut patterns for *Haemaphysalis* spp. of North America. **(A)** 16S PCR-RFLP cut patterns of *Haemaphysalis* spp. **(B)** *COI* PCR-RFLP cut patterns of *Haemaphysalis* spp.

The restriction enzymes for the PCR-RFLP were selected to optimize the differentiation of *H. longicornis* from other *Haemaphysalis* spp. in North America. The 16S PCR-RFLP was more reliable than the *COI* PCR-RFLP, as there were many more sequences available for comparison and the RFLP patterns were unique between species (Table 2, **Figure 5**). However, regardless of gene target, *H. longicornis* was effectively differentiated from other *Haemaphysalis* spp. native to North America. Unfortunately, sequence data for only one specimen of *H. chordeilis* was made available for analysis, so there is a possibility that there may be more than one PCR-RFLP cut pattern for this species for both the 16S and *COI* gene targets (**Figure 5**). Although this is a limitation for this molecular key, *H. chordeilis* is an avian specialist and is only rarely reported in the United States (29). The sequence of *H. chordeilis* was excluded in both gene analyses from clades including the other two native North American species (*H. juxtakochi* and *H. leporispalustris*) which may be explained based on previous morphologic work (Figures 1, 2) (47, 48). Although there is taxonomic debate regarding the validity of all *Haemaphysalis* subgenera *H. chordeilis* and *H. punctata* are both in the subgenus *Aboimisalis*, whereas *H. leporispalustris* and *H. juxtakochi* are both in the subgenus *Gonixodes* (47–49). Also, based on morphologic characteristics, *H. chordeilis* and *H. punctata* are very similar and may be difficult to differentiate. The addition of the 16S and *COI H. chordeilis* sequence data from this study provides valuable insight into the taxonomic position of this species. Furthermore, analysis of the PCR-RFLPs indicates that these closely related and morphologically similar *Haemaphysalis* spp. can be effectively differentiated with the *COI* PCR-RFLP (Figures 4A, 5B).

The RFLP patterns of *H. longicornis* were indistinguishable from a few *Haemaphysalis* species outside of North America (Figures 3A, 4A). However, *H. longicornis* can be distinguished from all of the species with available sequence data if both gene targets are included in the analysis. For the 16S PCR-RFLP, *H. longicornis* shared a cut pattern with *H. asiatica* which is endemic to Thailand and the surrounding countries and, thus far has, only been described to be infesting felids and canids native to that region (Figure 3A) (50). It is likely that the range of *H. longicornis* and *H. asiatica* overlap so further optimization of this assay within this region is warranted before use. We also detected more interspecies overlap in the 16S PCR-RFLPs, likely due to the larger amount of available sequence data. For the *COI* PCR-RFLP, there was less available sequence data for PCR-RFLP comparisons. One of the two *H. longicornis* cut patterns was shared with *H. flava* which is endemic to eastern Asia and infests a wide range of mammalian hosts including those utilized by *H. longicornis*, most notably sheep, cattle, and horses (Figure 4A) (41, 51).

*Haemaphysalis* is a highly speciose genus, being the second largest after *Ixodes* (52). Our study provided new sequence data from several *Haemaphysalis* species including *H. chordeilis* and the invasive *H. longicornis*. Specifically, for *H. leporispalustris* (rabbit tick) that is native to North America, we noted considerable sequence variation (92–99%) and multiple RFLPs for the *COI* gene target, which was unexpected because the intraspecific variation noted for other *Haemaphysalis* spp. with numerous sequences available rarely resulted in several unique RFLP patterns [e.g., *H. longicornis* and *H. qinghaiensis*; Tables 2, 3; (53, 54)]. This highlights the need for additional sequencing of individual ticks from different populations and host species to further evaluate the intraspecific genetic variability of different gene targets and the utility of the PCR-RFLPs described in this study. In summary, the 16S PCR-RFLP performed better than the *COI* PCR-RFLP as *Haemaphysalis* spp. sequences from North America and other countries had lower intraspecific variation, and there were a larger number of 16S sequences available on GenBank for comparison, for this reason only a key to species was generated for the 16S PCR-RFLP assay. While further optimization is warranted in other regions of the world where a higher diversity of *Haemaphysalis* spp. ticks of human and veterinary importance are present, the methods detailed here provide a faster and more cost-effective alternative to sequencing damaged tick specimens, especially those from North America. Finally, this study can serve as the foundation for similar, more region-specific PCR-RFLP molecular keys for the *Haemaphysalis* spp. and other vector species of one health importance.

## Key to *Haemaphysalis* Ticks Found in North America, Based on *DraI* Restriction Digestion of the 16S rRNA Gene Amplified by PCR (Figure 5A)

**Table:** 

1	More than two bands present………………………………………. 2
	Only two bands present………………………………………………. 3
2(1)	Largest band > 200 bp………………………………………………… 4
	Largest band <200 bp………………………………………………… 5
3(1)	Largest band > 400 bp…………………………………….…*H. leporispalustris*
	Largest band <300 bp………………………………………….…*H. chordeilis*
4(2)	Middle band approximately 200 bp…………………………*H. leporispalustris*
	Middle band approximately 175 bp…………………………….…*H. juxtakochi*
5(2)	Middle band at 160 bp…………………………………………….…*H. juxtakochi*
	Top band at 160 bp……………………………………………….…*H. longicornis*

## Data Availability Statement

The datasets generated for this study can be found in the NCBI GenBank: MN661147-MN661151, MN663150-MN663156, MN991269, and MN994495.

## Author Contributions

AT, KD, and MY designed and developed the assays and wrote the paper. AT developed the molecular key, analyzed final datasets, and created the figures and tables. CC, SD, KD, RF, TG, PI, LL, JL, TM, CO, RR-V, MR, DS, SV, and SW were integral in the collection of specimens for analysis. All authors contributed to manuscript revision, read and approved the submitted version.

### Conflict of Interest

The authors declare that the research was conducted in the absence of any commercial or financial relationships that could be construed as a potential conflict of interest.
